# The neural underpinnings of cognitive and postural profile of a young adult with congenital cerebellar athrophy: a longitudinal case report

**DOI:** 10.3389/fnins.2026.1724744

**Published:** 2026-02-05

**Authors:** Maria Devita, Adele Ravelli, Chiara Ceolin, Marina De Rui, Lorenzo Pini, Matteo Bendini, Michela Sarlo, Giuseppe Sergi, Umberto Castiello, Daniela Mapelli, Chiara Begliomini

**Affiliations:** 1 Department of General Psychology (DPG), University of Padua, Padua, Italy; 2 Geriatrics Unit, Department of Medicine (DIMED), University of Padua, Padua, Italy; 3 Department of Neurobiology, Care Sciences and Society, Karolinska Institutet and Stockholm University, Aging Research Center, Stockholm, Sweden; 4 Department of Neuroscience, University of Padua, Padua, Italy; 5 Padova Neuroscience Center, University of Padua, Padua, Italy; 6 Department of Neuroradiology, Treviso Hospital, AULSS 2, Treviso, Italy; 7 Department of Communication Sciences, Humanities and International Studies, University of Urbino Carlo Bo, Urbino, Italy

**Keywords:** case report, cerebellar ataxia, cerebellar disease, cognitive symptoms, computational morphometry, postural balance

## Abstract

For decades the cerebellum has been conceived as an organ chiefly involved in motor control, gait and posture. However, more recent and increasing evidence shows that the cerebellum is also involved in cognition and affection, and that damage to this structure may significantly compromise the overall neuropsychological functioning. Here we report we report the evolution along time of a possible congenital cerebellar atrophy and its effects on gross motor and cognitive functioning in a 41-years old man. Advanced neuroimaging analyses are also provided in order to understand the anatomo-functional correlates of cerebellar damage, with a novel focus on its different lobules. It is hypothesized that congenital cerebellar atrophy, initially compromising motor control, gradually brings to cognitive frailties. Findings related to neuroimaging, neuropsychological and postural data reveal a progressive decline in cognitive and gross motor capacities, particularly affecting executive and attentional functions. Neuroimaging results suggest specific gray matter volume reductions within cerebellar regions known to influence cognitive processes. This case report provides a nuanced look into the neuropsychological impact of cerebellar atrophy over time, shedding light on the elusive cognitive role of the cerebellum and its potential link to cerebellar cognitive affective syndrome.

## 1 Introduction

Long considered solely responsible for motor functioning, the cerebellum is now recognized for its critical role in various cognitive and affective functions (Schmahmann, 2004). Growing body of evidence indicates that cerebellar damage can lead to impairments across a wide range of neuropsychological domains, collectively referred to as Cerebellar Cognitive Affective Syndrome [CCAS, (Schmahmann and Sherman, 1998)]. CCAS presents deficits in executive function, memory, visuospatial processing, language, and emotional regulation, often more debilitating than the motor impairments traditionally associated with cerebellar dysfunction (Argyropoulos et al., 2020). Despite these advancements, the cerebellum’s precise role in cognitive processes remains enigmatic. The cerebellar contributions to cognition challenges established neural models and underscores its connectivity with higher-order cortical areas, conventionally linked to cognitive and emotional regulation (Schmahmann, 1991). However, how cerebellar atrophy–especially congenital forms–affects the trajectory of cognitive functions remains underexplored. Understanding the impact of cerebellar atrophy on cognitive functioning over time could help to decode the its contributions to human cognition and its possible role in neurodevelopmental and neurodegenerative conditions. This case report contributes to this field by exploring the longitudinal evolution of a young adult with congenital cerebellar atrophy. Through comprehensive cognitive, postural, and neuroimaging evaluations at baseline and a 2-years follow-up, this investigation provides a valuable opportunity to examine the differential roles of cerebellar lobules in motor versus cognitive functions.

## 2 Case description

A 41-years-old male patient (LS) was referred to our Neuropsychological Unit due to persistent difficulties with gait and posture. Ten years earlier, he had presented with headache, gait disturbances, visual difficulties, and left-sided dysmetria. A neuroradiological evaluation (magnetic resonance - MR) revealed an expansive lesion in the endo-sellar region, attributable to pituitary adenoma. Ophthalmic assessment confirmed a left hemifield deficit, along with left esotropia and diplopia. The MRI also revealed, for the first time, significant cerebellar atrophy which was labeled as “congenital” but was neither further investigated nor followed up. Despite this, the patient continued to report balance difficulties, which he perceived as progressively worsening over time. As for the patient’s history, he was married with two adolescent sons. He had completed high school and had worked for several years as a waiter. In recent decades, he had started his own business and served as president of a football club. Overall, he exhibited a medium level of cognitive reserve (Nucci et al., 2012).

## 3 Diagnostic assessment

### 3.1 Neuropsychological assessment

#### 3.1.1 Clinical interview

Speech was fluent and globally appropriate in form and content. The patient reported postural instability and visual disturbances (diplopia and nystagmus, corrected with prismatic lenses). He expressed concern about being perceived as “drunk” by others due to his gait and reported feeling burdened by these visual and motor limitations, which he always strived to hide. LS described himself as a compliant person, respected and appreciated by everyone. No memory or attention disturbances were reported. His account of personal history was slightly confused in the chronological sequencing of events. Spatial and temporal orientation were adequate. Mood and behavior were appropriate and stable during evaluation. Mild psychomotor slowing and atypical grip when holding the pen were observed.

#### 3.1.2 Neuropsychological and psychometric examination

A global screening test [Montreal Cognitive Assessment – MoCA; (Nasreddine et al., 2005)], selected tests from a comprehensive neuropsychological battery [Esame Neuropsicologico Breve 2 - ENB-2; (Mondini et al., 2011)], and measure of executive frontal functions [FABit; (Appollonio et al., 2005)] were administered (Table 1). LS’s cognitive profile was overall within normal limits (MoCA). The altered score on the Tangled Figures Test (ENB-2) was not considered clinically relevant, likely reflecting the aforementioned visual impairments. Concrete thinking emerged in language and executive function tests (Abstraction Test and Token Test). Although performance on these tests fell below normative thresholds, it was interpreted as indicative of LS’s reasoning style, a trait-like feature rather than a clinically significant neuropsychological deficit. LS demonstrated good self-monitoring throughout the assessment, and performance in other cognitive domains was broadly intact.

**TABLE 1:** Baseline and follow-up (after 2 years) neuropsychological and postural assessment.

Panel A – neuropsychological assessment						
Test	Score t_0_	Cut-off	Outcome t_0_	Score t_1_	Cut-off	Outcome t_1_
**Montreal Cognitive Assessment (MoCA)**						
Visuospatial/executive	5/5			4/5		
Naming	3/3			3/3		
Memory and attention	5/6			3/6		
Language	2/2			2/2		
Phonemic fluency	0/1			0/1		
Abstraction	2/2			2/2		
Delayed recall	4/5			3/5		
Orientation	6/6			6/6		
MoCA total score	RS = 27/30 AS = 26.15/30 ES = 4	15.5	On range	RS = 23/30 AS = 22.15/30 ES = 3	15.5	On range
Mini-Mental State Examination (MMSE) estimated by MoCa	29	24	On range	29	24	On range
**Esame Neuropsicologico Breve-2 (ENB-2)**						
Digit Span	5/8	5	On range	5/8	5	On range
Short story recall - immediate	16/28	8	On range	15/28	8	On range
Short story recall - delayed	21/28	11	On range	23/28	11	On range
Memory with interference – 10 sec	7/9	6	On range	9/9	6	On range
Memory with interference – 30 sec	8/9	4	On range	7/9	4	On range
Trial making test - A	47′′	55′′	On range	61′′ 2 err.	55	IMPAIRED
Trial making test - B	127′′	142′′	On range	133′′	142	On range
Token test	4.5/5	5	IMPAIRED	4.5/5	5	IMPAIRED
Verbal fluency (letter)	8	10	IMPAIRED	8	10	IMPAIRED
Abstract reasoning test	3/6	4	IMPAIRED	3/6	4	IMPAIRED
Cognitive estimation task	5/5	4	On range	5/5	4	On range
Tangled figure test	24	32	IMPAIRED	31	32	IMPAIRED
Drawing copy test	2/2	2	On range	2/2	2	On range
Drawing test	2/2	2	On range	2/2	2	On range
Clock test	10/10	8	On range	10/10	8	On range
Praxis	6/6	6	On range	6/6	6	On range
ENB-2 global score	71.96	77.19	IMPAIRED	73.19	77.19	IMPAIRED
**Frontal assessment battery (FAB)**						
Similarities (conceptualization)	3/3			3/3		
Verbal fluency (mental flexibility)	3/3			2/3		
Motor series (programming)	2/3			3/3		
Conflicting instructions (sensitivity to interference)	3/3			2/3		
Go–No Go (inhibitory control)	3/3			3/3		
Prehension behavior (environmental autonomy)	3/3			3/3		
FAB global score	RS = 17/18 AS = 15.9/18 ES = 3	≤13.4	On range	RS = 16/18 AS = 14.9/18 ES = 2	≤13.4	On range
**Cognitive Reserve Index Questionnaire (CRIq)**						
CRIq total score	101	84–114	Medium			Medium
CRI-Education			102			
CRI-Working Activity			112			
CRI-Leisure Time			101			
**Panel B – postural assessment**						
**General stability index results**						
	**Baseline (t0)**		**Follow-up (t1)**			
	**Observed score (STD dev.)**		**Observed score (STD dev.)**			
Postural stability index	1.0 (0.75)		1.2 (0.93)			
Anterior/posterior index	0.8 (0.78)		1.0 (0.95)			
Medial-lateral index	0.3 (0.30)		0.5 (0.38)			
**Limits of stability test results (LOS)**						
	**Baseline (t0)**		**Follow-up (t1)**			**Target**
**Directional control (DC)**	**Observed score**		**Observed score**			
General	51		46			65
Forward	44		22			65
Backward	80		86			30
Left	52		58			65
Right	62		87			65
Forward/to the left	48		44			65
Forward/to the right	51		52			65
Backward/to the left	71		59			65
Backward/to the right	51		91			65
**Fall risk test results**						
	**Baseline (t0)**		**Follow-up (t1)**			
	**Observed score (STD dev.)**		**Observed score (STD dev.)**			
	Interrupted due to difficulties to stand on the platform		4.1 (3.78)			

RS = raw score; AS = adjusted score, according to correction grids of Italian normative data; ES = equivalent score (0–4); CUT-OFF = threshold value of 5% worst obtained from a reference sample of healthy people referred to as normative sample. On range = the performance is quantitatively on range as expected according to age and education (above 5° percentile rank of the normative sample). IMPAIRED = the performance is equal or lower than the 5° percentile rank of the normative sample. Tests from: MoCA: Nasreddine et al., 2005; in Italian, Conti et al. (2015), Santangelo et al. (2015) MMSE (estimated by MoCA, Roalf et al., 2013): normative value in Magni et al. (1996). ENB-2: normative value in Mondini et al. (2011). CRIq: Nucci et al., 2012 (http://cri.psy.unipd.it). FAB: normative value in Appollonio et al. (2005).

### 3.2 Neuroimaging assessment

LS underwent MR evaluation on a 3T scanner Siemens VIDA at the Neuroradiology Department of Treviso Hospital (Italy). High-resolution structural T1-weighted images of the entire brain were collected (isotropic voxel of 0.9 mm^3^, repetition time (TR) = 2200 ms, echo time (TE) = 2.53 ms, flip angle (FA) = 8°, field of view (FOV) 260 mm × 260 mm). Diffusion Weighted Imaging (DWI) data were acquired using a single-shot spin-echo EPI sequence with TR = 8500 ms, TE = 97 ms, FOV = 307.2 mm × 307.2 mm, and 60 interleaved slices (no gaps) with an isotropic voxel of 2.2 mm^3^. The maximum diffusion weighting was 3000 s/mm^2^, with no diffusion gradients (*b* = 0 s/mm^2^) and 64 diffusion-weighted images. Previous conventional neuroradiological assessment described significant cerebellar atrophy affecting both hemispheres (see Supplementary Figure 1).

Structural MRI data were analyzed using Voxel-Based Morphometry (VBM) with the Spatially Unbiased Atlas Template (SUIT) toolbox in MATLAB environment (Diedrichsen et al., 2009). VBM enables voxel-wise comparisons across brain volumes based on standardized anatomical measures (Ashburner and Friston, 2000). The pipeline involved cerebellum isolation by creating individual masks, with visual inspection and manual correction if needed. The isolated cerebellar volumes underwent segmentation into gray and white matter; gray matter was then normalized and resliced into SUIT space, with modulation via Jacobian determinants of deformations to correct for non-linear transformation distortions. Finally, the resulting image was smoothed with a 6 mm Full Width at Half Maximum (FWHM) Gaussian kernel. This procedure was applied to T1-weighted images of LS and of a control group (CG) of 20 right-handed healthy male subjects (Mean age: 38.7 years, SD: 6.4) belonging to the NIMH Healthy Volunteers Dataset (Nugent et al., 2023). Dataset and participants were selected to provide the best possible match to LS’s demographics and key MRI parameters for VBM (magnetic field strength, sequence, voxel size, slice thickness). Harmonization was not applied due to its region-specific effects in T1-weighted MRI and the caution required in cerebellar VBM analyses (Gebre et al., 2023).

Comparisons between LS and CG were conducted using the General Linear Model (GLM two-samples *t*-test, equal variance assumed) to identify potential differences in Gray Matter Volume (GMV). Total Intracranial Volume (TIV), obtained with the CAT12 toolbox (Gaser et al., 2022) was included as a covariate to rule out the possibility of results due to intersubjects’ variability in total brain size rather than to local differences. Statistical images were thresholded with a Family-Wise Error (FWE) corrected rate (*p* < 0.05, k ≥ 50) To validate the results, permutation tests were conducted using a two-samples *t*-test, comparing each single CG component against a group formed by the remaining CG components plus LS (Dickie et al., 2015).

**FIGURE 1:**
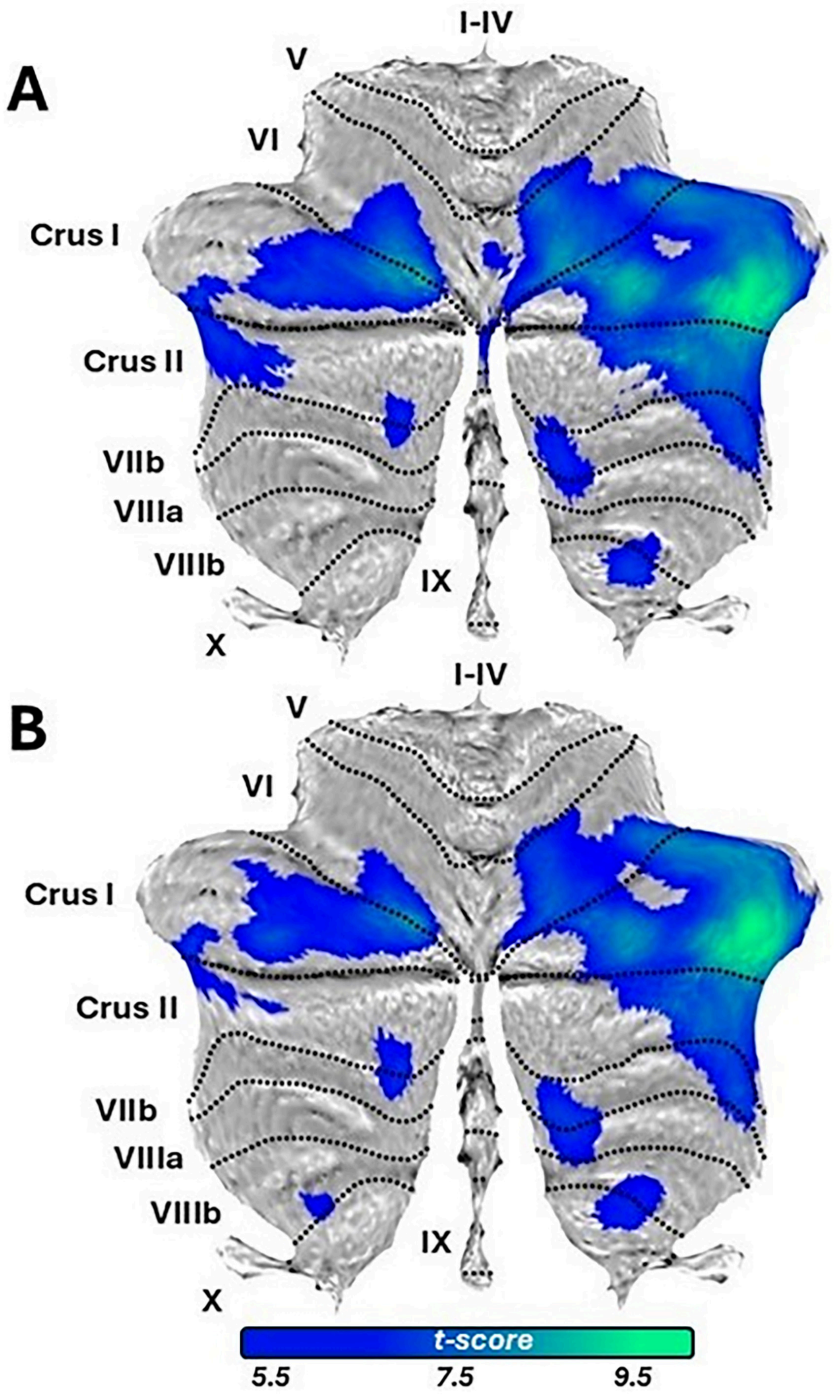
Gray Matter Volume (GMV) differences highlighted by the comparison CG > LS, for baseline and follow-up. The figure has been obtained with the SUIT toolbox (https://diedrichsenlab.org/imaging/suit.htm), overlaying the difference of GM images onto the flatmap template implemented in the toolbox. **(A)** Reports results for the baseline; **(B)** reports results for the 2 years follow-up.

The comparison LS > CG did not yield significant results, while the opposite comparison highlighted GMV major differences in Crus I bilaterally, and in Crus II to a minor extent. Additionally, lobule VI -both right side and vermis-, VIIB and IX showed significant differences (Figure 1A and Supplementary Table 1A).

### 3.3 Postural assessment

Postural and dynamic stability were evaluated using the Biodex Balance System [BBS; (Arnold and Schmitz, 1998; Clark et al., 1997; Hinman, 2000; Prometti et al., 2016; Table 2)]. The obtained scores, reflecting the body’s deviation from its center of mass, yielded an increased General Stability Index (GSI), suggestive of moderate instability. Dynamic balance, assessed through the Limits of Stability (LOS) test, showed reduced overall Directional Control (DC = 46), with a marked deficit in the forward direction (DC = 44), while rightward and backward movements were relatively preserved. Finally, in the Risk of Falling test, the patient was unable to maintain balance on the unstable platform, leading to an interruption of the task. Overall, these results indicate mild-to-moderate impairments in postural control, particularly affecting the forward plane, in line with the patient’s complaints of unsteady gait and balance difficulties.

**TABLE 2:** Baseline-follow-up GMV differences for LS (indicated in voxels).

Region	Baseline	Follow-up	Difference
Lobule I_IV_left	3548	3486	62
Lobule I_IV_right	4053	4191	−138
Lobule V_left	4660	4584	76
Lobule V_right	4381	4460	−79
Lobule VI_left	8994	8715	279
Lobule VI_right	7257	7107	150
Crus I_left	11656	11409	247
Crus I_right	10676	10295	381
Crus II_left	10324	10207	117
Crus II_right	9703	9602	101
Lobule VIIB_left	5152	5052	100
Lobule VIIB_right	4771	4660	111
Lobule VIIIA_left	5397	5043	354
Lobule VIIIA_right	4695	4368	327
Lobule VIIIB_left	4601	3841	760
Lobule VIIIB_right	4174	3618	556
Lobule X_left	818	739	79
Lobule X_right	857	794	63
Vermis VIIIB	732	616	116
Dentate_right	1787	1890	−103

Only differences >50 voxels are reported.

## 4 Follow-up (after 2 years)

### 4.1 Neuropsychological follow-up assessment

The same neuropsychological tests as in the previous evaluation were re-administered to assess LS’s cognitive profile over time (Table 1). The results revealed mild difficulties in sustained and selective attention, psychomotor speed (TMT-A), working memory (MoCA), and controlled lexical access (verbal fluencies). As in the earlier assessment, a tendency toward concrete thinking emerged in tasks tapping language and executive functions (Abstraction Test and Token Test, ENB-2). Overall, although LS’s cognitive performance remained within normal limits for his age and education, a subtle yet progressive decline was observed.

### 4.2 Neuroimaging follow-up assessment

**FIGURE 2:**
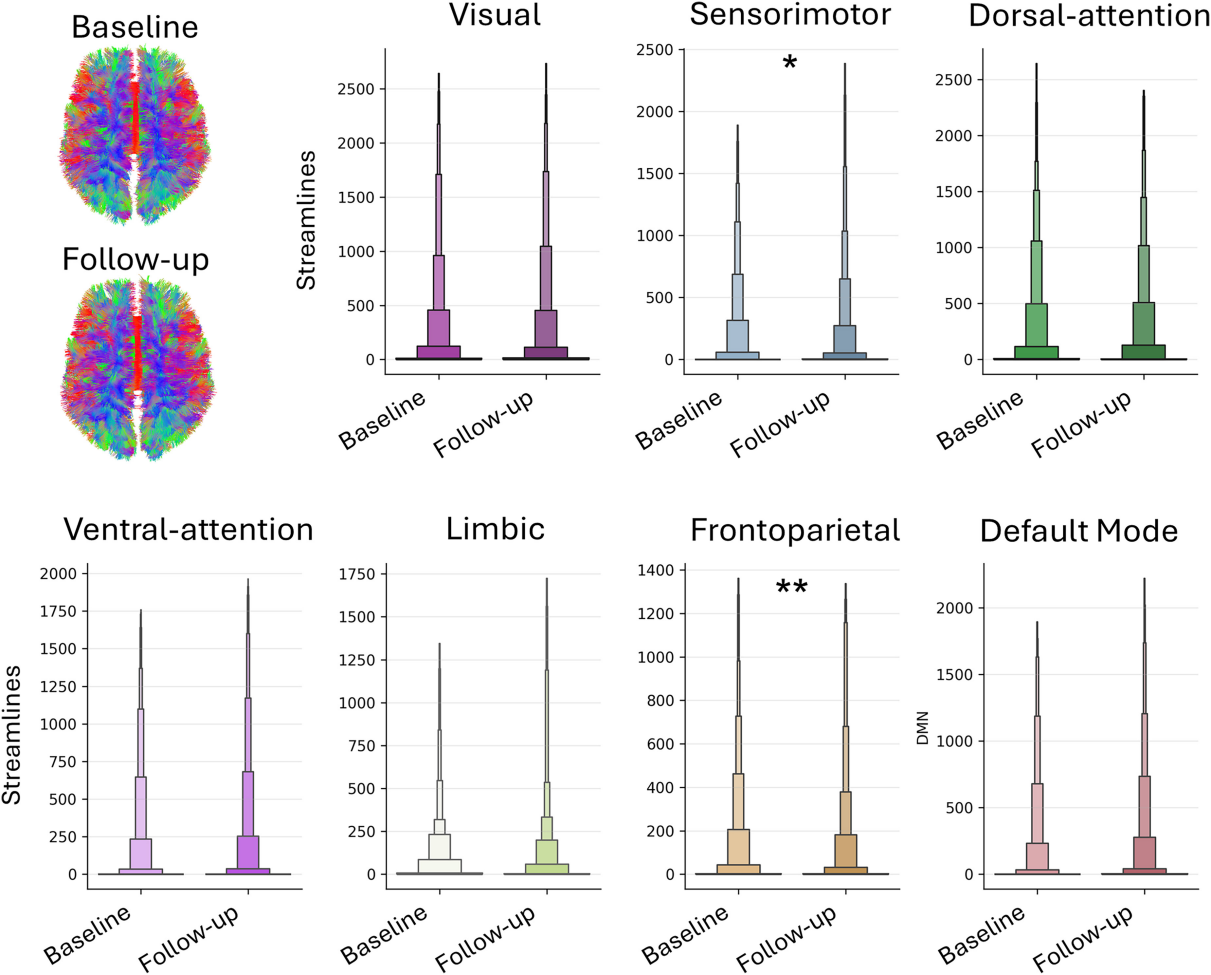
Network-wise longitudinal differences. Probabilistic tractography maps were generated from pre-processed diffusion-weighted imaging data collected for the two timepoints. Streamlines connecting parcels within the same network were compared with a non-parametric paired *t*-test, to assess within-network parcel-wise streamline differences after outlier removal. The corresponding within-network regions are overlaid on an inflated brain surface for visualization, maintaining a 1:1 color match with the network plots. Significant longitudinal differences are marked as ** (*p* < 0.01, Bonferroni corrected) and * (*p* < 0.05, Bonferroni corrected).

Structural T1-weighted MRI data were acquired and analyzed using the same parameters and procedures as at baseline. The LS > CG comparison yielded no significant results, mirroring baseline findings. The reverse comparison (CG > LS) revealed greater GMV differences in bilateral Crus I, with smaller differences in bilateral lobule VIIIB and right-sided lobules VI, VIIB, and IX (Figure 1B and Supplementary Table 1B). Permutation tests, like at baseline, detected no significant differences. A baseline-to-follow-up comparison was conducted for LS cerebellum structural images in order to qualitatively assess changes in lobular volume. The volume in terms of voxels contained in each individual lobule was obtained with SUIT. A Global GMV decrease was observed, mainly driven by bilateral reductions in VIIIA, VIIIB, Crus I, and VI, with smaller decreases in Crus II, VIIB, and X. Lobules I–IV and V showed mixed left-sided loss and right-sided gain; vermis VIIIB exhibited the largest loss. Conversely, cerebellar nuclei increased in volume, mainly in the right dentate nucleus (Figure 2, Table 2, and Supplementary material). Comparison between the two assessments was performed also on DWI data, adopting a probabilistic tractography model to compute the structural connectivity matrix on the images. Data were preprocessed as follows: (i) brain extraction and denoising using the MP-PCA [MRTrix3, (Veraart et al., 2016)]; (ii) local subvoxel-shifts method (Kellner et al., 2016) to reduce Gibbs ringing artifacts; (iii) motion, eddy currents correction, and removal of outlier slices [FSL, (Andersson and Sotiropoulos, 2016)]. Non-linear registration to the MNI space was applied to project Schaefer’s parcellation with *n* = 400 (Schaefer et al., 2018) to the native DWI space. Parcels were used as region-of-interest to compute the probabilistic tractography map. The structural connectome was defined as the streamline count between each pair of cortical nodes, which represents the number of white matter streamlines connecting the nodes. Fibers connecting parcels within the same network defined in Yeo’s atlas space with *n* = 7 (Yeo et al., 2011) were averaged. Within-network structural connectivity was compared through non-parametric paired test (Wilcoxon) to assess whole-network longitudinal differences. *P*-values < 0.05 Bonferroni-corrected (*n* = 7 networks) were considered significant. A decrease in streamlines connecting parcels within the frontoparietal network (*p* = 0.008) was observed (Figure 2), while a significant increase was detected for parcels connecting the sensorimotor network (*p* = 0.02). The remaining examined networks (visual, limbic, dorsal-attentional, ventral-attentional, and default mode) did not show results surviving multiple comparison correction. In conclusion, longitudinal analysis of brain morphometry and structural connectivity revealed structural alterations in specific cerebellar regions–particularly bilateral Crus I–as well as in frontoparietal connectivity in LS.

### 4.3 Postural follow-up assessment

At follow-up, LS showed poorer postural performance, particularly in the forward direction, which decreased from 44 to 22. In contrast, performance in the right and backward-right directions markedly improved (from 62 to 87 and from 51 to 91, respectively). The postural stability assessment confirmed this trend, revealing a decline over time in the forward directional control (DC) index, alongside improvements in rightward and backward-right control (Table 1). Recent evidence suggests that individuals with advanced cognitive decline perform worse than cognitively healthy peers in static balance tasks (general stability and fall risk tests), while under dynamic conditions their deficits are especially pronounced in directional control toward partial targets (Biasin et al., 2023). These difficulties may reflect both impaired motor coordination and planning due to cognitive deficits, as well as challenges in understanding task instructions.

## 5 Discussion

This case study of a young adult with congenital cerebellar atrophy offers significant insights into the cerebellum’s dual role in motor and cognitive functions. Across the longitudinal assessment period, progressive changes in cognitive profile and postural stability were observed, aligning with neuroimaging findings that highlighted structural deterioration in specific cerebellar regions known to support cognitive processes.

Cross-sectional comparisons revealed global cerebellar atrophy at both baseline and 2-years follow-up, primarily involving regions associated with the “cognitive” cerebellum [VI, Crus I, Crus II, VIIB, IX; (Saadon-Grosman et al., 2024; Stoodley and Schmahmann, 2009; Xue et al., 2021)]. The Crus, a key component of CCAS (Schmahmann and Sherman, 1998), together with lobule VIIB and, to a lesser extent, lobule IX (Saadon-Grosman et al., 2024), has been implicated in executive functioning and working memory deficits (Mariën et al., 2014; Ravizza et al., 2006; Stoodley and Schmahmann, 2010). The GMV atrophy observed in Crus I and II is notable, as these regions are closely connected to the prefrontal cortex and play a role in higher-order cognitive processes. Although this comparison should be interpreted cautiously for methodological reasons, the neuropsychological profile of LS (impaired language comprehension, verbal fluency, abstract reasoning, and visuospatial abilities) is consistent with reduced functionality in these regions. GMV atrophy at T0 affected lobule IX too, which is known for its role in balance and gait stability (Barmack and Yakhnitsa, 2021). Consistently, LS inability to maintain balance -both in daily life and during the Risk of falling test- may reflect the disruption of cerebellar mechanisms involved in anticipatory postural adjustments and dynamic equilibrium control, processes in which lobule IX plays a fundamental role. Differently, lesions to cerebellar lobule VI can lead to a mixed pattern of symptoms: cognitive impairment has been reported (Stoodley and Schmahmann, 2009), as well as timing issues, affecting limbs coordination (Schlerf et al., 2012). Lesions in this cerebellar region have been associated with upper limb ataxia (Manto et al., 2012), oculomotor deficits (Striemer et al., 2015), and motor learning difficulties (Bernard and Seidler, 2013), as well as affective disturbances, including emotional blunting and mood alterations (Schmahmann and Sherman, 1998). These findings are further supported by neuroimaging evidence (Guell et al., 2018; Wildgruber et al., 2005). Moreover, the connections of lobule VI with limbic and prefrontal regions (Devita et al., 2021; Manto et al., 2024) strengthen the hypothesis of the cerebellum as a key node in cortico-subcortical signaling pathways contributing to limbic circuitry.

More peculiarly, LS’s clinical evolution is consistent with GMV variations across cerebellar lobules and their functional specialization. At T1 LS cognitive profile was characterized by a worsening in attentional skills, psychomotor speed and verbal fluency, as well as in executive functioning, tracing a slight overall deterioration in comparison with T0. These cognitive changes are mirrored by GMV decreases mainly affecting cerebellum cognitive regions, such as Crus I and II, and VI to a minor extent (Devita et al., 2021; Guell et al., 2018; Stoodley and Schmahmann, 2009), and by diffusion tractography highlighting a significant decrease in structural connectivity within the frontoparietal network. This network is crucial for functions including attention, working memory, and cognitive control (Marek and Dosenbach, 2018), and decreased connectivity within it is typically observed in a variety of conditions where cognitive functioning is compromised, such as Alzheimer’s Disease (Dai et al., 2019) but also in multiple sclerosis (Rocca et al., 2016). Regarding postural control, at T1 LS showed an asymmetrical evolution characterized by emerging deficits in forward directional control on one side, contrasted by improvements in rightward and backward-right stability on the contralateral side. This pattern aligns with longitudinal GMV changes, including reductions in left lobules VI, VIIIA, VIIIB, and X, as well as vermal VIIIB, coupled with a relative GMV increase in the cerebellar nuclei, notably the right dentate. These regions integrate sensorimotor and vestibular signals for directional sway control, particularly forward stability, while vermal VIIIB coordinates axial balance and locomotion via brainstem projections (Conrad et al., 2025). GMV loss in these regions usually disrupts forward stability by impairing integration of vestibular and proprioceptive inputs for axial posture (Schlerf et al., 2012). Lobule X further modulates balance through eye-head coordination and vestibular processing, and the right dentate nucleus, with its output to premotor cortex, supports voluntary postural adjustments and limb coordination (Barmack, 2021). Together, these findings suggest that the forward-plane instability could be linked to regions critically involved in anticipatory postural adjustments and gait (Parrell et al., 2021), whereas the enhanced lateral and backward control may arise from compensatory recruitment of preserved lobular and nuclear circuitry (Schmahmann and Pandya, 2009). In this perspective, it is possible that processes of neuroplasticity led to the development of alternative motor strategies for movement control, a phenomenon not uncommon in individuals with cerebellar dysfunction (Deschamps et al., 2014). In this case, support to this view comes from the selective increase in structural connectivity observed within the sensorimotor network at T1.

LS longitudinal trajectory overall speaks in favor of cerebellar plasticity, where selective nuclear volume gains and sensorimotor network connectivity fosters compensatory postural strategies, despite cognitive deterioration, offering a model for adaptive reorganization in chronic cerebellar pathology. More broadly, these findings supports the cerebellum’s substantial contribution to cognitive and postural functions, suggesting that alterations within cerebellar regions can progressively erode both cognitive and motor capabilities, even if with different dynamics. These findings align with emerging research on CCAS, which posits that cerebellar atrophy can disrupt a broad range of cognitive and affective functions due to the cerebellum’s connectivity with cerebral cognitive networks (Schmahmann, 2019).

### 5.1 Limitations and future directions

Several limitations should be acknowledged when interpreting the present findings. As a single-case longitudinal observation, the study does not allow for generalization nor for formal statistical inference. Moreover, the clinical assessment relied primarily on cognitive and postural measures, without the inclusion of sensorimotor testing that could have provided a more direct link between changes in structural connectivity and motor performance. Methodologically, comparing data acquired from different scanners may introduce effects related to hardware differences. Despite the precautionary measures adopted, we cannot entirely rule out the presence of scanner-related effects. For this reason, results emerging from cross-sectional comparisons should be interpreted with caution. The absence of genetic testing represents an additional limitation, as hereditary ataxias cannot be formally excluded. Furthermore, the documentation available regarding the diagnosis of cerebellar atrophy was limited, and the classification of the condition as congenital was therefore based on retrospective clinical information and long-standing symptom history rather than on comprehensive developmental records.

Future investigations could build on these findings by systematically incorporating specific genetic testing and dedicated sensorimotor measures, as well as by developing rehabilitation protocols that combine cognitively demanding tasks with motor training specifically tailored to cerebellar atrophy. Finally, implementing regular, fine-grained neuroimaging follow-ups could improve close monitoring of possible structural changes and provide objective markers to refine and adapt intervention strategies over time.

## 6 Conclusion

This longitudinal single-case study provides unique insights into the cerebellum’s critical role in cognition and motor coordination. LS’ performance modifications are coherent with GMV reductions and connectivity disruptions in cerebellar regions associated with higher-order cognitive processes and postural stability. This case highlights the cerebellum’s substantial, though enigmatic, influence on cognitive health and posture, supporting the need for research into tailored interventions in individuals with cerebellar degeneration.

## Data Availability

The raw data supporting the conclusions of this article will be made available by the authors, without undue reservation.
