# Supplementary figures and images for: The neural underpinnings of cognitive and postural profile of a young adult with congenital cerebellar athrophy: a longitudinal case report
Source: Front Neurosci. 2026 Feb 5;20:1724744. doi: 10.3389/fnins.2026.1724744 (PMC12916574; doi:10.3389/fnins.2026.1724744)

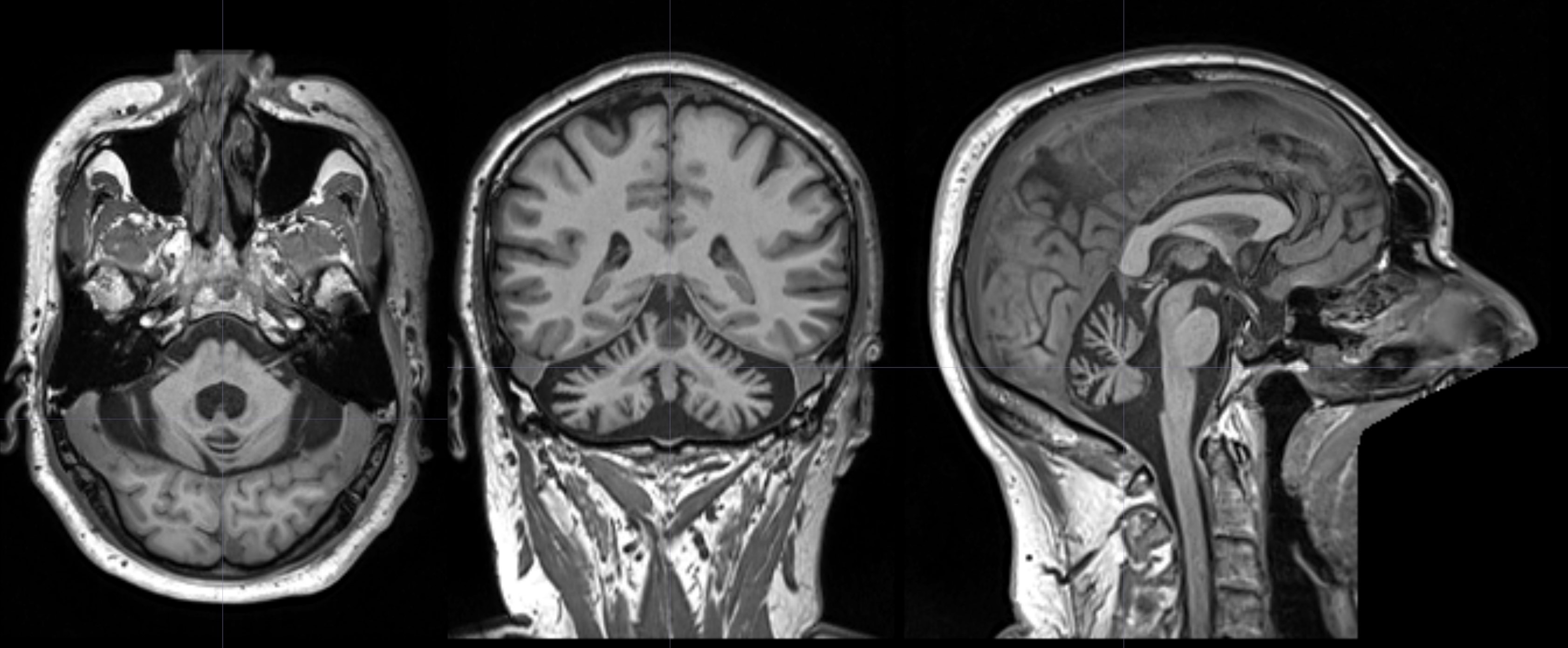

Supplement: Supplementary file 1 [file Image_1.jpeg]

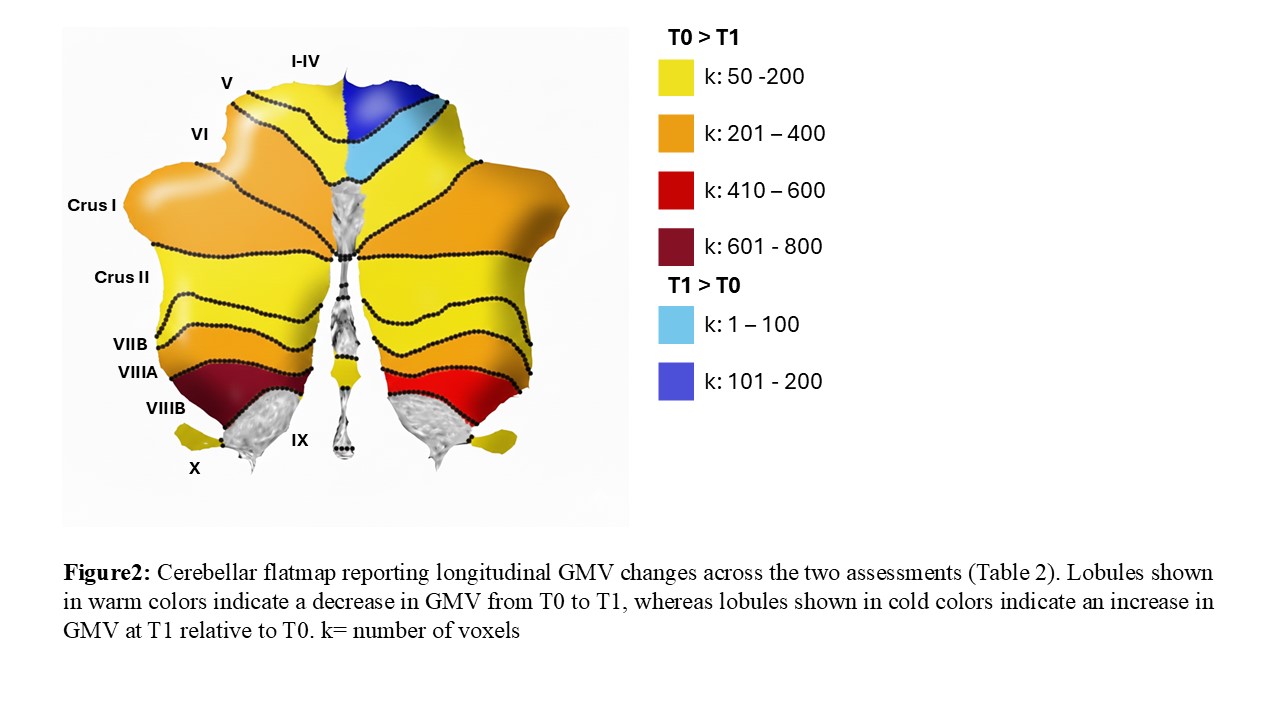

Supplement: Supplementary file 2 [file Image_2.jpg]
