# Supplementary material for: The neural underpinnings of cognitive and postural profile of a young adult with congenital cerebellar athrophy: a longitudinal case report
Source: Front Neurosci. 2026 Feb 5;20:1724744. doi: 10.3389/fnins.2026.1724744 (PMC12916574; doi:10.3389/fnins.2026.1724744)
Supplement: Supplementary file 3 [file Table_1.docx]

Supplementary Material

# Supplementary Figures and Tables

## Supplementary Tables

**Supplementary Table 1.** Results of the between-groups comparison (CG > LS) at the baseline. The following details are reported: k = number of voxels; t and z scores; p values (both uncorrected and FWE-corrected) stereotaxic coordinates according to the Montréal Neurological Institute (MNI); cerebellum side and lobule. Results were considered significant at FWE correction at cluster level (p < 0.05, k>100). Panel A reports baseline results, Panel B reports 2-years follow-up results.

| **Set-level** | | **Cluster-level** | | | **Peak-level** | | | | **MNI coordinates** | | | **Anatomical details** | |
| --- | --- | --- | --- | --- | --- | --- | --- | --- | --- | --- | --- | --- | --- |
| *p(unc)* | *Clusters* | *p(FWE)* | *k* | *p(unc)* | *p(FWE)* | *T* | *z* | *p(unc)* | *x* | *y* | *z* | *Side* | *lobule* |
| **PANEL A - baseline** | | | | | | | | | | | | | |
| **0.000** | **6** | **0.000** | **16410** | **0.000** | **0.000** | **9.190** | **5.529** | **0.000** | **40** | **-55** | **-39** | **right** | **CRUS I** |
|  |  |  |  |  | 0.001 | 8.334 | 5.270 | 0.000 | 17 | -75 | -23 | right | VI |
|  |  |  |  |  | 0.001 | 8.245 | 5.242 | 0.000 | 29 | -77 | -30 | right | CRUS I |
|  |  |  |  |  | 0.002 | 7.606 | 5.027 | 0.000 | 53 | -63 | -38 | right | CRUS I |
|  |  |  |  |  | 0,003 | 7.390 | 4.949 | 0.000 | 43 | -54 | -32 | right | CRUS I |
|  |  |  |  |  | 0.006 | 7.003 | 4.805 | 0.000 | 30 | -74 | -42 | right | CRUS II |
|  |  |  |  |  | 0.026 | 6.004 | 4.393 | 0.000 | 2 | -64 | -28 | right | VERMIS VI |
|  |  | **0.000** | **3954** | **0.000** | **0.001** | **8.407** | **5.293** | **0.000** | **-17** | **-77** | **-25** | **left** | **CRUS I** |
|  |  |  |  |  | 0.010 | 6.604 | 4.648 | 0.000 | -34 | -70 | -34 | left | CRUS I |
|  |  | **0.000** | **554** | **0.007** | **0.011** | **6.574** | **4.636** | **0.000** | **10** | **-48** | **-53** | **right** | **IX** |
|  |  | **0.001** | **380** | **0.021** | **0.013** | **6.454** | **4.586** | **0.000** | **-38** | **-50** | **-40** | **left** | **CRUS I** |
|  |  |  |  |  | 0.021 | 6.140 | 4.453 | 0.000 | -36 | -59 | -46 | left | CRUS II |
|  |  | **0.001** | **337** | **0.028** | **0.016** | **6.306** | **4.524** | **0.000** | **13** | **-69** | **-43** | **right** | **VIIB** |
|  |  | **0.004** | **206** | **0.076** | **0.022** | **6.112** | **4.440** | **0.000** | **-14** | **-78** | **-46** | **left** | **CRUS II** |
| **PANEL B – 2 years follow-up** | | | | | | | | | | | | | |
| **0.019** | **7** | **0.000** | **13688** | **0.000** | **0.000** | **9.356** | **5.576** | **0.000** | **47** | **-56** | **-40** | **right** | **CRUS I** |
|  |  |  |  |  | 0.001 | 8.152 | 5.212 | 0.000 | 44 | -53 | -33 | right | CRUS I |
|  |  |  |  |  | 0.001 | 7.897 | 5.127 | 0.000 | 53 | -62 | -38 | right | CRUS I |
|  |  |  |  |  | 0.004 | 7.304 | 4.918 | 0.000 | 50 | -61 | -32 | right | CRUS I |
|  |  |  |  |  | 0.004 | 7.215 | 4.885 | 0.000 | 17 | -74 | -23 | right | VI |
|  |  |  |  |  | 0.007 | 6.871 | 4.754 | 0.000 | 43 | -47 | -50 | right | VIIB |
|  |  |  |  |  | 0.032 | 5.877 | 4.336 | 0.000 | 25 | -62 | -26 | right | VI |
|  |  | **0.000** | **3057** | **0.000** | **0.001** | **8.088** | **5.190** | **0.000** | **-16** | **-78** | **-24** | **left** | **CRUS I** |
|  |  |  |  |  | 0.008 | 6.774 | 4.716 | 0.000 | -37 | -64 | -36 | left | CRUS I |
|  |  |  |  |  | 0.022 | 6.130 | 4.449 | 0.000 | -29 | -67 | -33 | left | CRUS I |
|  |  | **0.000** | **607** | **0.005** | **0.011** | **6.540** | **4.622** | **0.000** | **15** | **-68** | **-44** | **right** | **VIIB** |
|  |  | **0.005** | **183** | **0.092** | **0.013** | **6.442** | **4.582** | **0.000** | **-38** | **-51** | **-39** | **left** | **CRUS I** |
|  |  |  |  |  | 0.029 | 5.940 | 4.364 | 0.000 | -35 | -60 | -45 | left | CRUS II |
|  |  | **0.001** | **345** | **0.026** | **0.015** | **6.345** | **4.541** | **0.000** | **-13** | **-78** | **-46** | **left** | **VIIB** |
